# Low expression of miR-142-3p promotes intervertebral disk degeneration

**DOI:** 10.1186/s13018-020-02194-4

**Published:** 2021-01-14

**Authors:** Jianmin Xue, Baoyang Hu, Wenhua Xing, Feng Li, Zhi Huang, Wenkai Zheng, Bo Wang, Yong Zhu, Xuejun Yang

**Affiliations:** 1 Graduate School of Inner Mongolia Medical University, Hohhot City, 010059 Inner Mongolia China; 2 Department of Thoracolumbar Spine Surgery, The Second Affiliated Hospital of Inner Mongolia Medical University, No. 1 Yingfang Road, Hohhot City, 010059 Inner Mongolia China

**Keywords:** Intervertebral disk degeneration, miR-142-3p, Cell behavior

## Abstract

**Background:**

Intervertebral disk degeneration (IDD) is a degenerative disease characterized by cytoplasm loss and extracellular matrix degradation. Numerous evidence reported that miRNAs participated in IDD development. Nevertheless, the function of miR-142-3p in IDD development remains unknown. This study mainly explored the potential role and function of miR-142-3p in IDD development.

**Methods:**

One percent fetal bovine serum was used to induce the degeneration of ATDC5 cells, and miR-142-3p level was examined by qRT-PCR. Then, miR-142-3p mimic/inhibitor and its corresponding negative control were transfected into ATDC5 normal and degenerative cells. Viability, migration, invasion, apoptosis, cycle, Bax, Bcl-2, P62, and Beclin1 expression levels were assessed using CCK8, wound healing assay, annexin V-FITC/PI staining, western blot, and qRT-PCR, respectively.

**Results:**

The results revealed that the expression levels of MMP13, ADAMTS5, MMP3, and Col-X were increased as well as the expression levels of SOX-9 and Col-II were reduced in ATDC5 degenerative cells, indicating the degeneration model was constructed. We observed that miR-142-3p was decreased in ATDC5 degenerative cells and its suppression could promote ATDC5 cell degeneration. However, miR-142-3p overexpression could reverse the cell viability inhibition, as well as apoptosis and autophagy enhancement in ATDC5 degenerative cells.

**Conclusions:**

Our results proved that miR-142-3p may play an important role in disk degeneration. Further animal study is needed to illustrate the role of the miR-142-3p in IDD development.

**Supplementary Information:**

The online version contains supplementary material available at 10.1186/s13018-020-02194-4.

## Background

Low back pain severely affects people’s ability to work and their quality of life. Globally, 540 million people have long-term loss of mobility due to low back pain [1]. It is reported that low back pain is the leading cause among more than 300 diseases that cause human disability [2]. The data of the Global Burden of Disease Study 2016 indicated that 6.73 million individuals had low back pain in China [3]. A widely recognized contributor to low back pain is intervertebral disk degeneration (IDD), which is the pathological basis for the occurrence of diseases such as herniated disk, spinal stenosis, and spinal deformity. It is a degenerative disease characterized by cytoplasm loss and extracellular matrix degradation followed by morphological and biomechanical changes. IDD can lead to cervical and low back pain and/or radiation pain in the upper limbs and lower limbers. In severe cases, it can cause weakening of the muscles of the limbs and even paraplegia, which not only seriously affects life quality of the patients, but also poses a heavy social and economic burden. Therefore, it is crucial to explore the pathophysiological mechanism of IDD.

MicroRNAs (miRNAs) are small non-coding RNAs with regulatory function in eukaryotic cells [4]. It is reported that miRNAs regulate cell proliferation, differentiation, apoptosis, growth, and other physiological processes [5]. miRNAs play a crucial role in the pathogenesis of tendon injuries and osteoarthritis [6, 7]. For example, Oliviero summarized that the expression pattern of many miRNAs (miRNA 22, miRNA-27b, miRNA-485-5p, miRNA-634, and so on) was changed in osteoarthritis [6]. Giordano and colleagues indicated that miRNAs could regulate tendon stem/progenitor cell senescence and suppress collagen formation in human tendons, suggesting miRNAs has potential as a therapeutic target in tendon injuries [7]. And others reported that miR-146a inhibited osteoclastogenesis and that administration of miR-146a prevented joint destruction in arthritic mice [8]. These results highlighted the role of miRNAs in bone disease.

Furthermore, numerous studies in recent years suggested that miRNAs played a crucial role in IDD development via participating in the process of chondrocyte proliferation, apoptosis, and extracellular matrix synthesis [9, 10]. For example, Tan et al. covered that the miR-665 level was overexpressed in degenerative human samples and its expression level was positively correlated with the Pfirrmann grade [11]. Sun et al. have shown that knockdown of miR-499a-5p promoted nucleus pulposus (NP) cell apoptosis and downregulated aggrecan and collagen II [12]. Others also revealed that miR-210 expression was decreased in IDD patients compared with the control group [13]. In addition, miR-142-3p has been studied in osteoarthritis and system sclerosis in recent years [14, 15]. However, there were few reports on the regulatory effect of miR-142-3p on IDD.

Therefore, miR-142-3p mimic/inhibitor and negative control (NC) were transfected into ATDC5 cells in this study. The effect of miR-142-3p on cell viability, apoptosis, and autophagy-related protein was evaluated. This finding may provide possible therapeutic and diagnostic targets for IDD.

## Methods

### Cell culture and treatment

The ATDC5 cell was obtained from the BeNa Culture Collection (Suzhou, China). Cells were cultured in DMEM /F12 medium (Hyclone, USA) containing 10% fetal bovine serum (FBS, Hyclone, USA) in a humidified incubator at 37 °C with 5% CO_2_. In addition, ATDC5 cells were treated in 1% FBS (Hyclone, USA) to induce cell degeneration. Passages 4 to 10 of ATDC5 cells were used in the present study.

### Cell transfection

The expression plasmids of miR-142-3p mimic/inhibitor and NC were purchased from GenePharma Co. Ltd (Shanghai, China). They were transfected into ATDC5 cells using Lipofectamine 2000 Transfection Reagent (Thermo Scientific, USA) in accordance with the user’s protocol. The sequences were presented in Supplemental Table S1.

### Cell viability assay

We used cell counting kit-8 (CCK-8, MCE, USA) to measure viability. Firstly, cells were added to 96-well plates, followed by adding 10 μL of CCK-8 solution and incubating for 1 h. Then the absorbance at 450 nm was determined with Microplate Reader (BioTek, USA).

### Wound healing assay

Cell migration was tested by wounding healing assay. Cells were added to a 6-well plate and scratched with a sterile 200-μL pipette tip. Then, cells were cultured in serum-free medium at 37 °C with 5% CO_2_. Finally, a digital camera was used to acquire images of the scratches of the cells at 0, 24, 48, 72 h. Wound surface area was examined by ImageJ software.

### Cell apoptosis analysis

Cell apoptosis was analyzed using flow cytometry with annexin V-FITC Apoptosis Detection Kit (Keygen Biotech, China). In brief, cells were added to a 6-well plate and were trypsinized and then washed twice with PBS. Subsequently, cells were harvested and re-suspended with 500 μL binding buffer, followed by staining with 5 μL annexin V-FITC and 5 μL propidium iodide in the dark for 15 min. Finally, cells were observed and detected by flow cytometry (Beckamn, USA), and data was analyzed by the FlowJo software (Treestar, Ashland, USA).

### Cell cycle analysis

Cells were washed with PBS and fixed with 70% ethanol for 2 h. After fixation, cells were again washed with PBS then stained with 500 μL PI/ RNase A staining solution in the dark. Finally, cell cycle was measured using flow cytometry (Beckamn, USA).

### Transwell assay

Transwell assay was performed using Matrigel-coated Transwell chambers (24-well insert; 8 μm pore size, Corning, USA). Generally, cells were added to the transwell chamber and 15% FBS medium (Hyclone, USA) was added to the lower chamber. After Giemsa staining, the cell number from 5 different fields was counted at × 400 magnification microscope (Sunny Optical Technology, China).

### Quantitative reverse transcription PCR

To evaluate the mRNA level of Bax, Bcl-2, P62, Beclin1, MMP-3, MMP-13, ADAMTS5, Col-X, SOX-9, and Col-II, total RNA was extracted from cells using TRIzol (Sigma, USA). Reverse transcription and qRT-PCR were conducted by PrimeScrip^TM^ RT Master Mix (TAKARA, Japan) and TB Green^TM^ Premix Ex TaqII (Takara, Japan). Total miRNA was isolated by miRNeasy Mini Kit (Qiagen, China). Reverse transcription and qRT-PCR were performed by miRcute miRNA cDNA First-Strand Synthesis Kit (TIANGEN, Beijing, China) and miRcute miRNA qRT-PCR SYBR Kit (TIANGEN, Beijing, China), respectively. The internal genes were U6 and GAPDH. The relative gene expression was calculated by the 2^-Δ(ΔCt)^. The sequences for PCR primers are shown in Table 1.

**Table 1:** The primers for qRT-PCR analysis

Gene	Sequence
miR-142-3p	GCGCGTGTAGTGTTTCCTACTTTATGGA
U6-F	CTCGCTTCGGCAGCACA
U6-R	AACGCTTCACGAATTTGCGT
Bax-F	AGACAGGGGCCTTTTTGCTAC
Bax-R	AATTCGCCGGAGACACTCG
Bcl-2-F	GTGGATGACTGAGTACCTGAACC
Bcl-2-R	AGCCAGGAGAAATCAAACAGAG
P62-F	GTGGGACAGCCAGAGGAACA
P62-R	GCCCTTCCGATTCTGGCAT
Beclin-F	GGGTCACCATCCAGGAACTCA
Beclin-R	CACCATCCTGGCGAGTTTCA
SOX-9-F	GTGAAGAACGGACAAGCGGA
SOX-9-R	AGATTGCCCAGAGTGCTCG
LC3B-F	GCGCTTGCAGCTCAATGCTA
LC3B-R	GTACACTTCGGAGATGGGAGTGG
MMP-13-F	CTTCCTGATGATGACGTTCAAG
MMP-13-R	GTCACACTTCTCTGGTGTTTTG
MMP-3-F	TGTCACTGGTACCAACCTATTC
MMP-3-R	TCTCAGGTTCCAGAGAGTTAGA
ADAMTS5-F	CAGTTTGCCTACCGCCATTG
ADAMTS5-R	CCACATAGTAGCCTGTGCCC
Col-X-F	AACAGGTATGCCCGTGTCTG
Col-X-R	CCTACCCAAACGTGAGTCCC
Collagen II-F	CCCTGGAAGAGATGGTGCAG
Collagen II-R	GTGAAACCTCGGTGTCCCTT
GAPDH-F	AGGACTGGATAAGCAGGGCG
GAPDH-R	CTGGAACAGGGAGGAGCAGA

### Western blot

Total protein from cells was isolated using RIPA lysate (CWBio, China). Protein concentration was evaluated using Microplate Reader (BioTek, USA), and equivalent amounts of total protein were applied for each immunoblot. The total proteins were electrophoresed in 10% Tris-glycine sodium dodecyl sulfate-polyacrylamide gels. After electrophoresis, protein was transferred to 0.45 μm polyvinyl difluoride membranes (PVDF, Roche, Switzerland), which were then blocked with 5% skim milk for 2 h. Blots were subsequently incubated with the primary antibody. The PVDF membranes were then incubated with secondary antibodies for 2 h at 37 °C, and the protein lane was shown by chemiluminescence using ECL Substrate (Bio-Rad, USA). Quantification of band intensity was performed with the ImageJ software. The antibodies used are shown in Supplemental Table S2.

### Statistical analysis

Data are presented as mean ± standard error of mean (SEM). Statistical analysis was performed by one-way analysis of variance (ANOVA). Differences were deemed significant at *p* < 0.05.

## Results

### The degeneration model was constructed

**Fig. 1:**
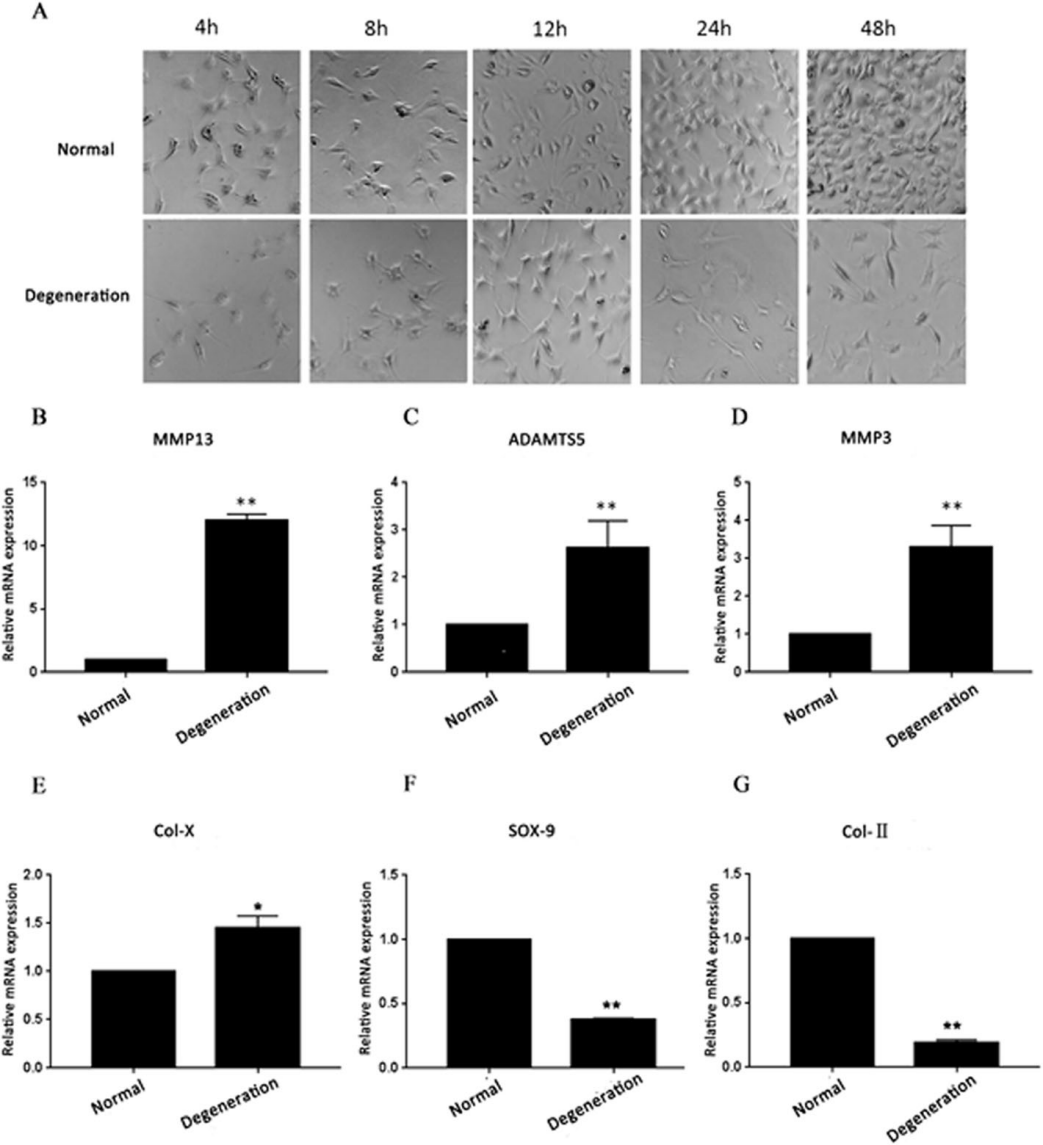
1% FBS induced the degeneration of ATDC5 cells. **a** The morphological changes of ATDC5 cells were observed using a microscope. The mRNA levels of **b** MMP13, **c** ADAMTS5, **d** MMP3, **e** Col-X, **f** SOX-9, and **g** Col-II. *p < 0.05, **p < 0.01 vs. normal cells

In this study, 1% FBS was used to treat ATDC5 cells to induce degeneration. To determine whether the degeneration model was successfully established, morphological changes and extracellular matrix-related gene expression were detected. It is reported that matrix metalloproteinase (MMP) and a disintegrin and metalloproteinase with thrombospondin motifs (ADAMTSs) can degrade extracellular matrix (ECM) components, including matrix membrane collagen, mesenchymal collagen, fibrin, and various proteoglycans [16–18]. Type X collagen (Col-X), type II collagen (Col-II), and SRY-type high mobility group box 9 (SOX-9) played a critical role in chondrogenesis [19, 20]. As shown in Fig. 1a, ATDC5 cells became slender after 1% FBS treatment compared with normal cells. Then, qRT-PCR analysis revealed that 1% FBS treatment increased the mRNA levels of MMP13 (Fig. 1b, *p* < 0.01), ADAMTS5 (Fig. 1c, *p* < 0.01), and MMP3 (Fig. 1d, *p* < 0.01), Col-X (Fig. 1e, *p* < 0.05) and reduced the mRNA levels of SOX-9 (Fig. 1f, *p* < 0.01) and Col-II (Fig. 1g, *p* < 0.01). These results suggested that the ATDC5 degenerative cell model was successfully established.

### miR-142-3p was downregulated in degenerative cells

**Fig. 2:**
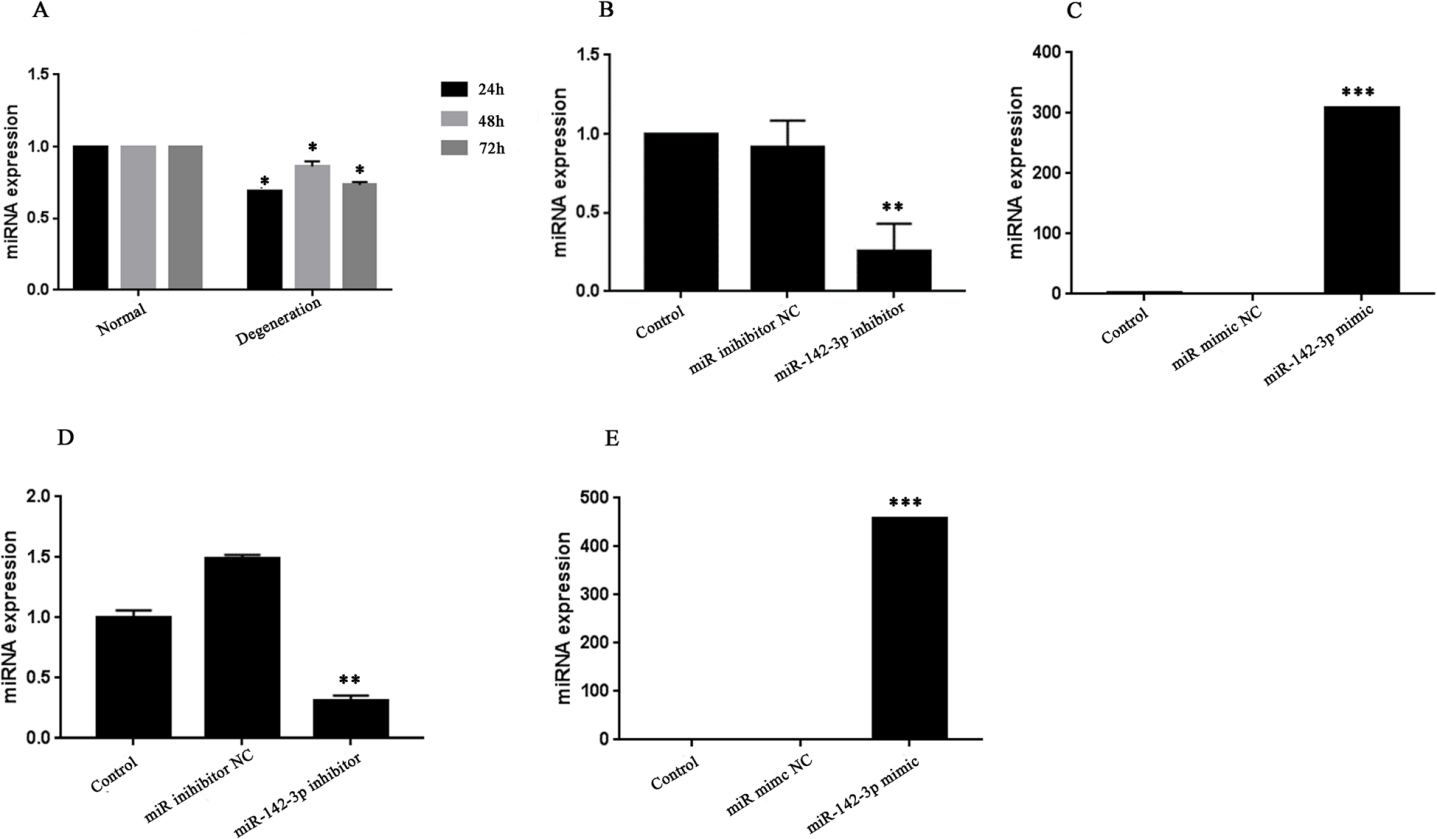
The mRNA level of miR-142-3p in ATDC5 cells. **a** MiRNA-142-3p was downregulated in degenerative cells. **b**, **c** The mRNA expression of miR-142-3p in normal cells after transfection of miR-142-3p inhibitor/mimic, respectively. **d**, **e** The mRNA expression of miR-142-3p in ATDC5 degenerative cells after transfection of miR-142-3p inhibitor/mimic. *p < 0.05 vs. normal cells, **p < 0.01, ***p < 0.01 vs. the corresponding NC group

Firstly, miR-142-3p expression was detected by qRT-PCR. As exhibited in Fig. 2a, the mRNA level of miR-142-3p was remarkably reduced in ATDC5 degenerative cells. The results demonstrated that miR-142-3p may be involved in ATDC5 cells degeneration.

### Decreased expression of miR-142-3p promoted cell apoptosis and autophagy in normal cells

**Fig. 3:**
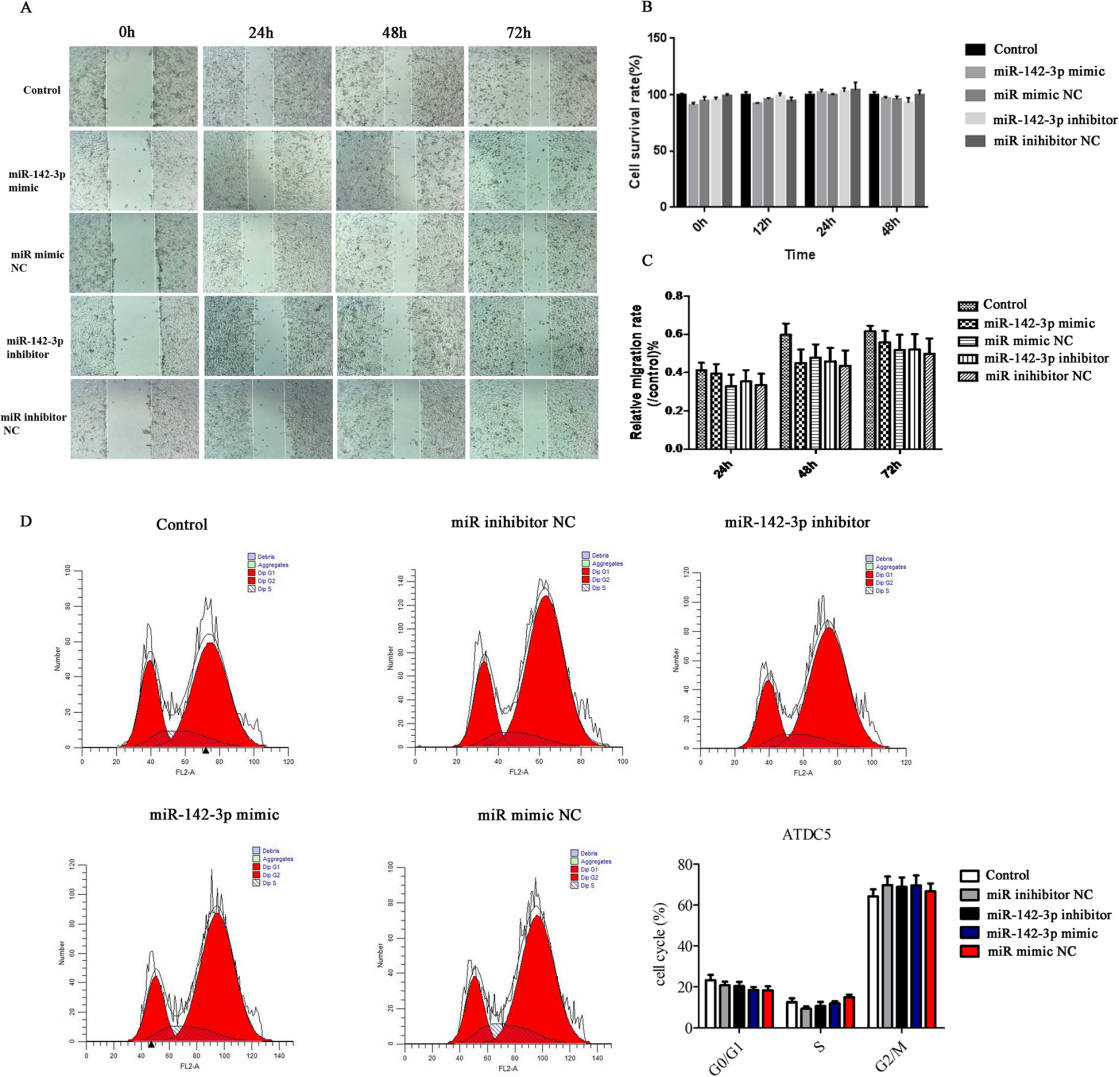
The effect of miR-142-3p on viability, migration, and cycle. **a**, **c** The migration rate was detected using wound healing assay. **b** The cell viability was assessed by CCK8. **d** Cell cycle analyzed by flow cytometer

**Fig. 4:**
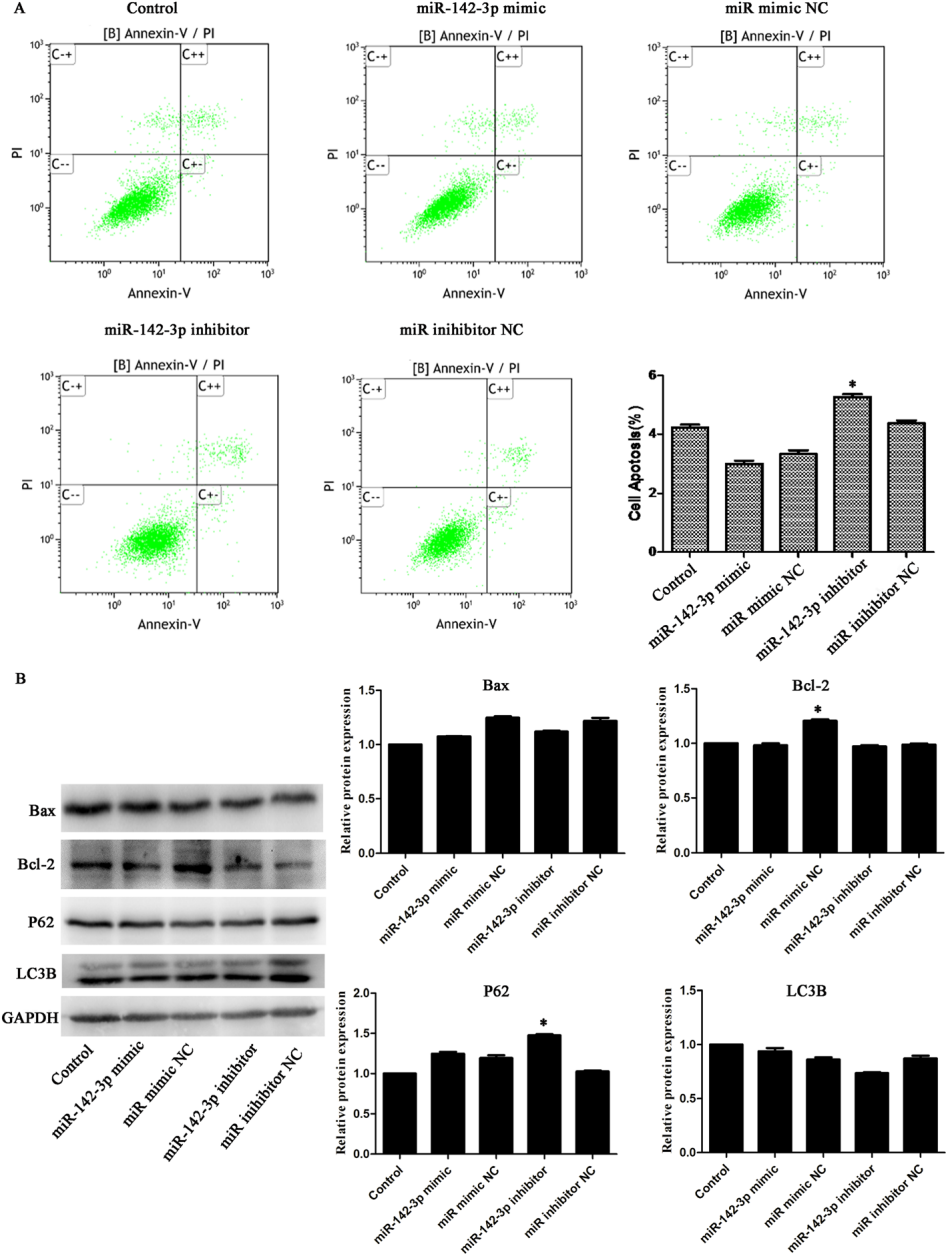
Decreased expression of miR-142-3p promoted apoptosis in normal cells. **a** Cell apoptosis analyzed by flow cytometer. **b** Protein levels of Bax, Bcl-2, P62, and LC3B were assessed using western blot. *p< 0.05 vs. the corresponding NC group

We examined the effect of aberrant miR-142-3p on normal cells, and then miR-142-3p mimic/inhibitor and NC were transfected into normal cells. Figure 2b, c presented that the transfection was successful. As exhibited in Figs. 3a–d and 4b, there was no significant difference in viability, migration, cell cycle, and the expression of Bax and LC3B among the five groups (*p* > 0.05). However, Fig. 4a, b displayed that apoptotic cells and P62 protein level were increased after inhibitor transfection (*p* < 0.05). These results proved that miR-142-3p downregulation could facilitate apoptosis and autophagy in normal cells.

### Overexpression of miR-142-3p inhibited the degeneration of cell

**Fig. 5:**
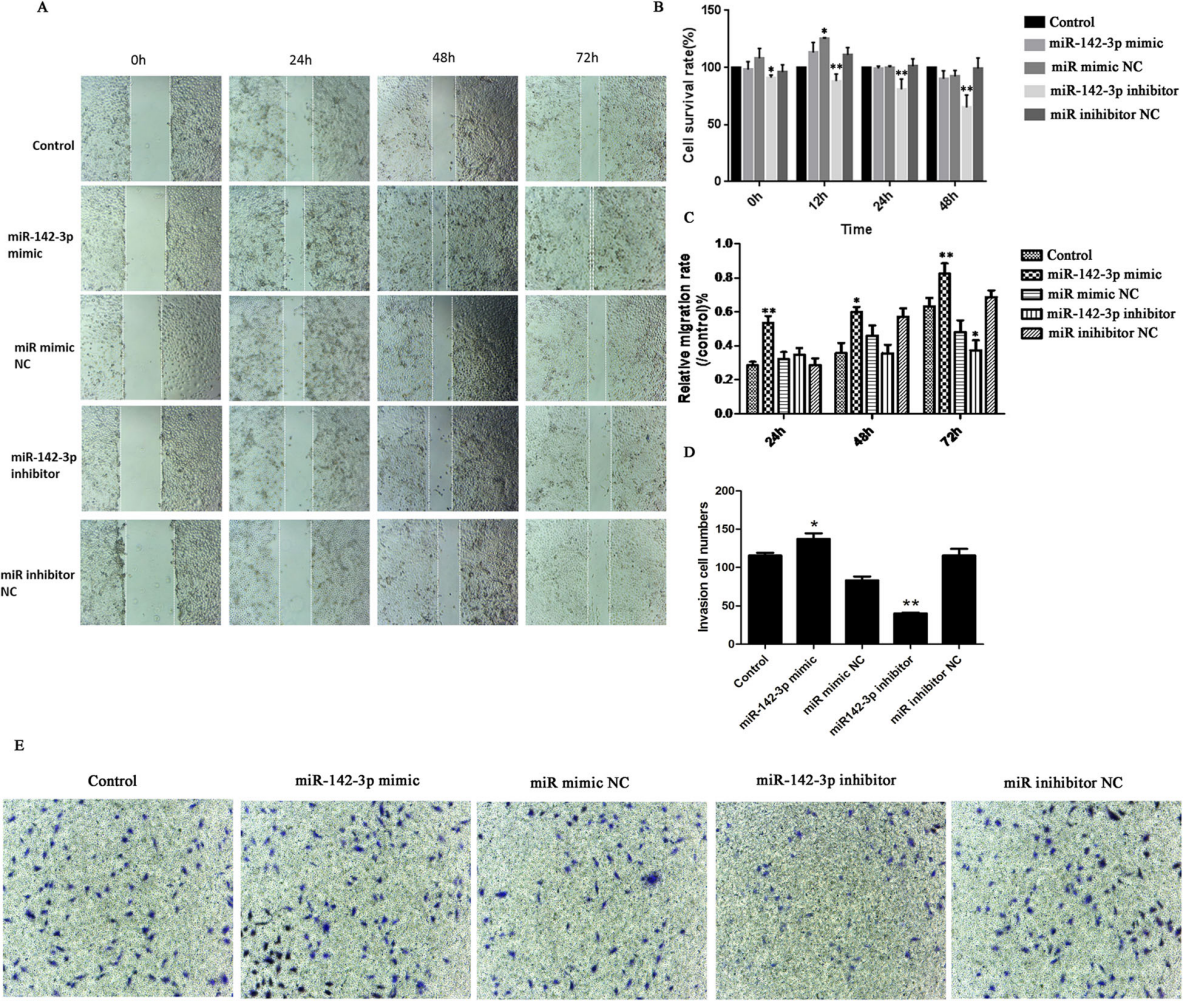
Overexpression of miR-142-3p promoted viability, migration, and invasion in degenerative cells. **a**, **c** Cell migration, **b** viability, and **d**, **e** invasion were detected by CCK8 assay, wound healing assay, and Transwell assay. *p < 0.05, **p < 0.01 vs. the corresponding NC group

**Fig. 6:**
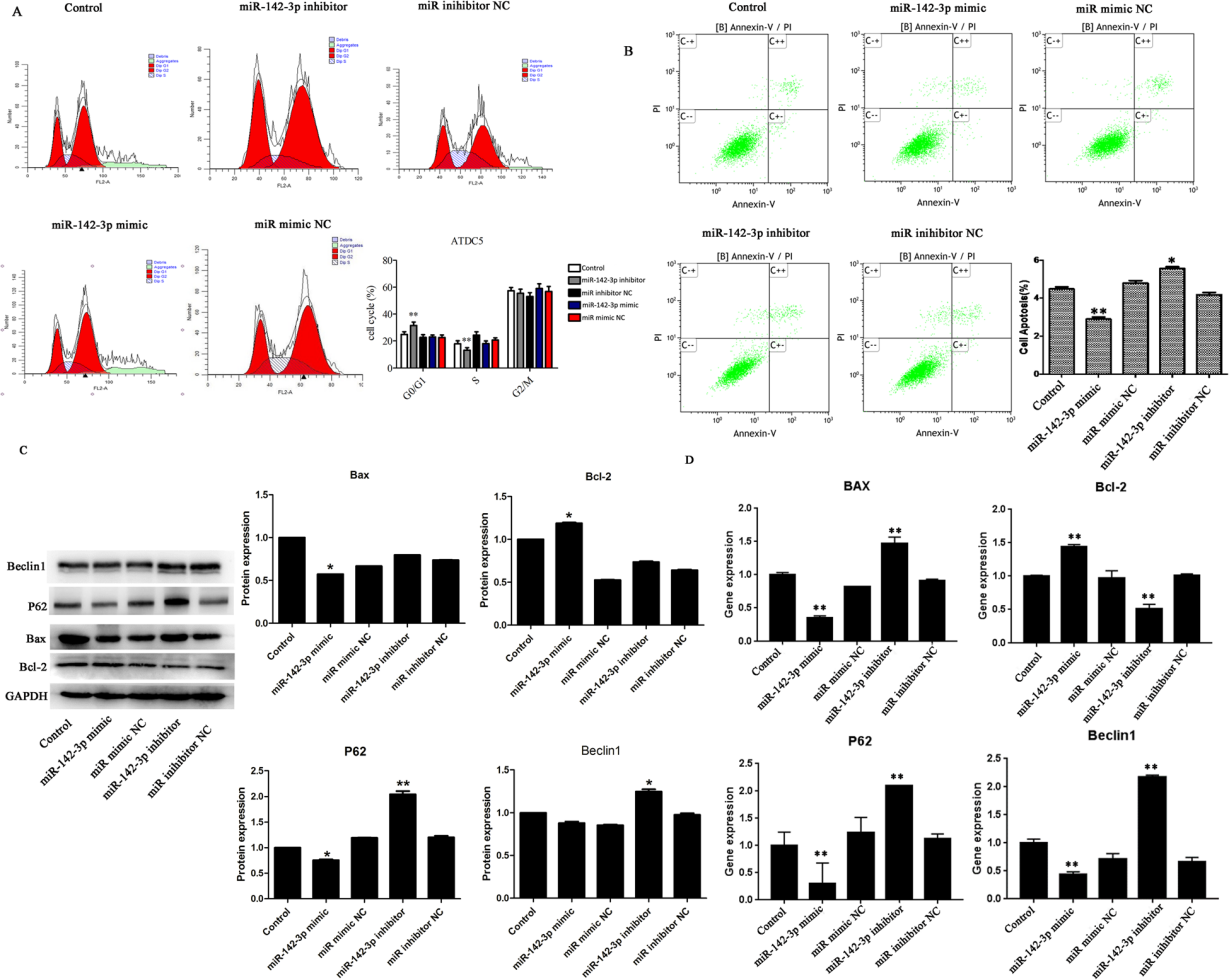
Upregulation of miR-142-3p reversed cell degeneration. **a** Cell cycle, **b** apoptosis, **c** protein, and **d** mRNA levels of Bax, Bcl-2, P62, and Beclin1 were assessed in ATDC5 degenerative cells. *p< 0.05, **p< 0.01 vs. the corresponding NC group

To explore the effect of miR-142-3p on cell degeneration, miR-142-3p mimic/inhibitor were transfected into degenerative cells. After transfection, PCR analyses verified the effectiveness of both miR-142-3p mimic and inhibitor in manipulating miR-142-3p expression (Fig. 2d, e). As exhibited in Figs. 5a–e and 6b, downregulation of miR-142-3p reduced viability, migration, and invasion, as well as promoted apoptosis in degenerative cells (*p* < 0.05 or *p* < 0.01). Besides, Figure 6a showed that inhibition of miR-142-3p increased cells in the G0/G1 phase and decreased cells in the S phase (*p* < 0.01). Western blot analysis showed that the protein levels of P62 and Beclin1 were upregulated in the miR-142-3p inhibitor group (Fig. 6c, *p* < 0.01or *p* < 0.05). Figure 6d also illustrated that the mRNA levels of Bax, P62, and Beclin1 were overexpressed, while Bcl-2 mRNA level was downregulated in the miR-142-3p inhibitor group (*p* < 0.01). Collectively, these data further demonstrated that downregulation of miR-142-3p promoted cell degeneration.

In addition, Figs. 5a–e and 6b displayed that upregulation of miR-142-3p increased cell viability, migration, and invasion and suppressed apoptosis in degenerative cells (*p* < 0.05 or *p* < 0.01). Furthermore, Fig. 6c, d presented that the protein and mRNA levels of Bax, P62, and Beclin1 were downregulated while Bcl-2 were upregulated after mimic transfection (*p* < 0.05 or *p* < 0.01). These results suggested that miR-142-3p overexpression can prevent the degeneration of cells.

## Discussion

IDD is the most common disease in spinal surgery and can cause a series of spinal disorders including disk herniation, discogenic pain, spinal stenosis, spinal segmental instability, degenerative scoliosis, and spondylolisthesis. In this study, we observed that the miR-142-2p level was reduced in degenerative cells. Overexpression of miR-142-3p could facilitate cell viability, migration, and invasion and inhibit apoptosis and autophagy in degenerative cells. These data uncovered that the abnormality of miR-142-3p might be involved in IDD development.

miRNAs play a crucial regulatory role in cell proliferation and differentiation, tissue growth, cell apoptosis, signal transduction, and other life activities [21]. It is demonstrated that miRNAs are essential for the maintenance of cellular function through fine-tuning the expression of multiple target genes [6]. miRNA210 may positively regulate osteoblastic differentiation by suppressing the TGFβ/activing signaling pathway by inhibiting activating A receptor type 1B [22]. Li et al. illustrated that miRNA506 inhibited rheumatoid arthritis fibroblast-like synoviocytes proliferation and induced apoptosis by targeting Toll-like receptors [23].

In recent years, miRNAs have attracted more and more attention to the development of IDD [24, 25]. Tao et al. suggested that miR-92 mRNA level was enhanced in IDD samples and promoted the proliferation of degenerated NP cells by targeting ARID2 [26]. In addition, Wang et al. reported that miR-154 was increased in NP cells of IDD patients, and inhibition of miR-154 could weaken the degeneration of IDD by increasing Col-II and aggrecan [27]. Evidence showed that miR-141 facilitated the apoptosis of NP cells by regulating SIRT1, thereby accelerating the degeneration of IDD [28]. Nevertheless, the function of miR-142-3p in IDD development has not been studied. In our study, we found that the expression level of miR-142-3p was decreased in degenerative cells. Similarly, our results further indicated that suppression of miR-142-3p could facilitate the degeneration of cells. However, miR-142-3p overexpression can reverse the degeneration of cells. These data proved that miR-142-3p could participate in the pathogenesis of IDD.

Additionally, the role of miR-142-3p has been studied in other diseases. For example, Qiang et al. found miR-142-3p was overexpressed in patients with rheumatoid arthritis and inhibition of miR-142-3p reduced the cell viability and increased apoptosis rate through regulating NF-κB signaling pathway [29]. Another study has demonstrated that miR-142-3p suppressed chondrocyte apoptosis and inflammation in osteoarthritis by targeting HMGB1 [14]. These findings demonstrated that miR-142-3p may be involved in the occurrence and development of diseases via interacting with other molecules or pathways. However, we only performed the basic research that miR-142-3p affect the biological function of IDD in this study. And more researches are needed to clarify the exact mechanism of miR-142-3p interact with other molecules to participate in IDD development.

## Conclusions

In summary, these data suggested that miR-142-3p may play an important role in disk degeneration. Further animal study is needed to fully illustrate the role of the miR-142-3p in IDD development.

## Supplementary Information

**Additional file 1: Table S1.** Sequences of miR-142-3p mimic, inhibitors and negative control. **Table S2.** Antibodies used in this study.

## Data Availability

All data generated or analyzed during this study are included in this published article.
