# Supplementary material for: Low expression of miR-142-3p promotes intervertebral disk degeneration
Source: J Orthop Surg Res. 2021 Jan 14;16:55. doi: 10.1186/s13018-020-02194-4 (PMC7809750; doi:10.1186/s13018-020-02194-4)
Supplement: Supplementary file 1 — Additional file 1: Table S1. Sequences of miR-142-3p mimic, inhibitors and negative control. Table S2. Antibodies used in this study. [file 13018_2020_2194_MOESM1_ESM.docx]

Table S1 Sequences of miR-142-3p mimic, inhibitors and negative control

| Variable | Sequence |
| --- | --- |
| miR-142-3p inhibitor | UCCAUAAAGUAGGAAACACUACA |
| miR-142-3p mimic | UGUAGUGUUUCCUACUUUAUGGAC  AUAAAGUAGGAAACACUACAUU |
| miR-142-3p inhibitor-NC | CAGUACUUUUGUGUAGUACAA |
| miR-142-3p mimic-NC | UUCUCCGAACGUGUCACGUTT |

Table S2 Antibodies used in this study

| Antibodies | Source |
| --- | --- |
| rabbit anti-P62 | Proteintech |
| rabbit anti-LC3B | Proteintech |
| rabbit anti-Beclin1 | Proteintech |
| rabbit anti-Bax | Proteintech |
| rabbit anti-Bcl-2 | Proteintech |
| mouse anti-GAPDH | Proteintech |
| goat HRP-anti-rabbit IgG | Proteintech |
| goat HRP-anti-mouse IgG | Proteintech |
